# Exploration and Gas Source Localization in Advection–Diffusion Processes with Potential-Field-Controlled Robotic Swarms †

**DOI:** 10.3390/s23229232

**Published:** 2023-11-16

**Authors:** Patrick Hinsen, Thomas Wiedemann, Dmitriy Shutin, Achim J. Lilienthal

**Affiliations:** 1 Institute of Communications and Navigation, German Aerospace Center (DLR), 82234 Wessling, Germany; thomas.wiedemann@dlr.de (T.W.); dmitriy.shutin@dlr.de (D.S.); 2 Chair of Perception for Intelligent Systems, School of Computation, Information and Technology (CIT), Technical University of Munich (TUM), 80992 Munich, Germany; achim.j.lilienthal@tum.de

**Keywords:** swarm robotics, robotic exploration, uncertainty mapping, artificial potential field control, gas exploration, gas source localization, advection–diffusion equation

## Abstract

Mobile multi-robot systems are well suited for gas leak localization in challenging environments. They offer inherent advantages such as redundancy, scalability, and resilience to hazardous environments, all while enabling autonomous operation, which is key to efficient swarm exploration. To efficiently localize gas sources using concentration measurements, robots need to seek out informative sampling locations. For this, domain knowledge needs to be incorporated into their exploration strategy. We achieve this by means of partial differential equations incorporated into a probabilistic gas dispersion model that is used to generate a spatial uncertainty map of process parameters. Previously, we presented a potential-field-control approach for navigation based on this map. We build upon this work by considering a more realistic gas dispersion model, now taking into account the mechanism of advection, and dynamics of the gas concentration field. The proposed extension is evaluated through extensive simulations. We find that introducing fluctuations in the wind direction makes source localization a fundamentally harder problem to solve. Nevertheless, the proposed approach can recover the gas source distribution and compete with a systematic sampling strategy. The estimator we present in this work is able to robustly recover source candidates within only a few seconds. Larger swarms are able to reduce total uncertainty faster. Our findings emphasize the applicability and robustness of robotic swarm exploration in dynamic and challenging environments for tasks such as gas source localization.

## 1. Introduction

Accurately predicting how airborne substances spread over space and time is of critical importance for disaster response efforts in the context of incidents involving chemical, biological, radiological, or nuclear materials. Localizing the source of a substance escaping into the environment becomes interesting when considering harmful or toxic gases, unpleasant odors, or chemical compounds of geological origin. Examples of such applications include early warning systems for chemical plants, monitoring of pipelines and industrial facilities, tracking the unwanted release of greenhouse gases from landfills as well as surveying of geological activities on remote celestial bodies. Utilizing mobile robotic platforms equipped with suitable sensors and which are capable of autonomous operation emerges as an outstanding solution for safely navigating and operating within these hazardous or difficult environments.

To improve the autonomy of these robotic systems, it is essential to enable them to gain an understanding of their surroundings. This perception and awareness can in turn be leveraged in the development of their operational and control strategies. From the engineering perspective, this can be achieved either with data-driven learning methods, or by incorporating domain knowledge, such as a gas dispersion model provided by physics. The latter is particularly attractive when we consider non-visual sensors, producing limited (as compared to visual sensors, like cameras) amounts of data. This approach forms the basis of the methodology discussed in this research.

The general problem we consider in our work is referred to as *gas source localization* (GSL) [1] in the robotic olfaction community. It concerns itself with identification of releases of gaseous or airborne chemical compounds, and more specifically with the detection and identification of the locations of these sources. When a gas is released, an increase in concentration in the local surroundings of the source will be the immediate result. Fluid dynamic effects like advection (wind) or diffusion may then carry on the increased concentrations further into the environment. By observing this process through measurements of gas concentrations in adequately chosen locations in the environment, one can try to infer the location of the gas release.

Although some of these tasks can conceivably be conducted by human surveyors, robotic systems can bring clear advantages. They eliminate the need to expose workers to potentially harmful substances, and allow access to otherwise inaccessible or hostile environments. We view GSL tasks as particularly well suited to *multi-robot systems*, i.e., teams of gas-detecting rovers or drones working in tandem to solve the source localization problem. Such a system can benefit from the individual platform’s high mobility, the robustness to failure of single robots, the possibility of intelligent cooperative data processing approaches, but most importantly from a significantly increased spatial sampling rate of the gas dispersion process, as compared to that achievable by a single surveyor. Various research groups in the field have developed experimental robotic platforms for the purpose of GSL, be that on the lab scale [2,3,4] or as full-scale robotic platforms [5]. While some focus on the robotic aspects alone, many aspire to develop machines that can operate as a swarm. The key question that arises in this context is how to control a multi-agent system in a way that is beneficial for GSL.

Early robotic GSL techniques were quite restrictive, assuming a fixed number of sources and integrating source estimation into the robot’s movement strategy. For example, *chemotaxis*-based [6,7] and *anemotaxis*-based [8,9] approaches aimed to guide robots along chemical concentration gradients. However, these methods faced limitations due to the complexity of real chemical plumes, in particular, smoothness of the concentration gradient [10]. More promising approaches in this context explore probabilistic methods that utilize the inherent mathematical properties of the dispersion process. Rather than tracking gas concentration gradients, these techniques treat the sources as hidden parameters that are then inferred based on the gathered data [11]. Bayesian methods for parameter estimation [12,13] are particularly attractive in this context. They enable what is typically referred to as *infotaxis* approaches [14], where information about the parameters of interest, e.g., the precision of source release rate estimates, is used to define objectives for autonomous navigation. These objectives aim to enhance GSL and guide the robots in finding the sources using different information-theoretic criteria (see, e.g., [14,15,16]).

This article presents a further development of infotaxis-based GSL for multi-robot systems. We extend here our previous work [17], where we proposed a potential-field-based method [18,19,20] for navigating a swarm of robots toward locations with high expected information gain. The model used in [17] is a simplified description of the gas propagation based on Poisson’s equation. In essence, this model assumed a steady state model of gas propagation driven only by diffusion from an unknown number of sources. Although simple, it allowed us to derive a spatial distribution that quantifies information, and use this directly as a potential function for navigation of our multi-robot system, while avoiding collisions and deadlocks. This goes in contrast to approaches where the potential function needs to be explicitly constructed by first determining discrete target locations from the information metric [21]. In this extension paper, we go beyond this highly idealized model of gas dispersion and consider the effects of advection as well as time dependency of the gas propagation. To this end, we use a model based on an advection–diffusion equation driven by an unknown source distribution. Through a probabilistic relaxation of the model, we then formulate and sequential inference algorithm that allows for computing both instantaneous concentration and source distributions, as well as the corresponding uncertainties. The latter are used to compute an artificial potential function for navigating the robots towards areas of high information content. We validate the method in simulations, demonstrating its ability to efficiently localize the sources with a swarm of mobile robots.

The following paper is structured as follows. We will begin in Section 2.1 by introducing the process model that is used to describe the gas dispersion. Next, in Section 2.3 we derive a probabilistic framework that will lead to the *uncertainty map*. It encodes a spatial informativeness of all possible future measurement locations. For the robots introduced in Section 2.4, and building upon this map, a potential-field-based control scheme is developed in Section 2.5 that guides the swarm agents to informative locations. In Section 3, we will discuss the simulation system as well as the experiment settings used to validate the method. This section is followed by a detailed analysis of the simulation results and an assessment of the suitability of the developed exploration strategy and control scheme. Finally, in Section 4 and Section 5 we discuss and summarize our findings and provide an outlook for future work.

### Notation

Throughout this paper we will make use of the following notation. Vectors are represented as boldface lowercase letters, e.g., x, and matrices as boldface uppercase letters, e.g., X. Their transpose is denoted by ${\left(\;\cdot \;\right)}^{\;T}$. For a square matrix X the expression diag(X) defines a vector composed of elements on the diagonal of X. We denote the probability density function (PDF) of a Gaussian random vector with expectation a=E{x} and covariance matrix $\Sigma =E\{\left(x-a\right){\left(x-a\right)}^{\;T}\}$ as N(x|a,Σ). We will use the notation $\delta_{x}\left(\Omega \right)$ to specify a (multidimensional) Dirac measure over domain Ω with a support at x∈Ω. We introduce the operator $\propto^{e}$ to indicate proportionality in the log domain; in other words, $x\propto^{e}y$ implies that x=const·exp(y) for some constant term const independent of *y* and *x*.

## 2. Methods

### 2.1. Process Model

At the core of the gas exploration strategy, a model of the underlying process physics is required, describing how the gas released from the source is transported into the environment. At the most general level, the dynamics of the propagating material can be described by the Navier–Stokes equations [22]. When some fluid-dynamical effects can be neglected, e.g., the compressibility of the material, a simplified descriptions can be chosen. In this work, we specifically focus on the advection–diffusion equation. We assume incompressible, isothermal flow of gas in a two-dimensional exploration environment $\Omega \subset R^{2}$ without convection. The mechanism of advection will describe the transport of the material with the wind $v\left(t\right)\in R^{2}$, which is assumed to be homogeneous over the exploration area. The total release volume is assumed to be minor, such that the total volume and pressure are unchanging, and the released gas exhibits neutral buoyancy. The continuous-time continuous-space rendition of the corresponding advection–diffusion partial differential equation (PDE) is then given as

(1) $$\begin{array}{cccc} \; & \frac{df(x,t)}{dt}=\kappa \;\Delta f\left(x,t\right)-v^{\;T}\left(t\right)\;\nabla f\left(x,t\right)+q\left(x\right),\; & \; & x\in \Omega ,\;t\in R_{+}, \end{array}$$

(2) $$\begin{array}{cccc} \; & s.t.\;f(x,t)=0,\; & \; & x\in \Gamma ,\;t\in R_{+}, \end{array}$$

where the time-varying concentration field f(x,t) over the spatial coordinate $x\;=\;{\left(x,\;y\right)}^{\;T}\;\in \;R^{2}$ is excited by the source distribution q(x). Diffusion—the first summand on the right-hand side of (1)—is parameterized by the diffusion coefficient κ. The second summand resprents the advection term, followed by the excitation that models gas sources. An important part of the model is the constraint (2), which specifies the Dirichlet boundary condition over the boundary $\Gamma \subset R^{2}$.

Although in some cases analytical solutions to (1) can be found [23], numerical solutions are often of necessary. To this end, the process model is discretized, e.g., using the finite difference method (FDM). Specifically, we partition Ω into *N* cells, from which *Q* discrete cells represent the interior of Ω excluding the boundary. The concentration in a cell is assumed to be constant, thus the continuous functions f(x,t) and q(x) can be represented as *N*- and *Q*-dimensional discrete vectors f(t) and q, respectively. Note that per our boundary condition (2), there can be no sources on Γ; thus, $q\in R^{Q}$. To discretize time, we introduce the discretization time interval ΔT, and sample time as t=kΔT, $k\in N_{0}$. Now, skipping some details on discretization (the interested reader is referred to [22] for an excellent introduction into finite difference and finite element methods), we can transform Equations (1) and (2) into

(3) $$\begin{array}{cc} \; & I_{Q\times N}\;\frac{f_{k}-f_{k-1}}{\Delta T}=\kappa \;L\;f_{k}-v_{k}^{\;T}\;D\;f_{k}+q, \end{array}$$

(4) $$\begin{array}{cccc} \; & B\;f_{k}=0,\; & \; & k\in N_{0}, \end{array}$$

where $L\in R^{Q\times N}$ is a discretized Laplace operator, $D\in R^{2\times Q\times N}$ is a tensor expressing a finite differences gradient for wind in 2D, and $I_{Q\times N}$ is a selection matrix that extracts the *Q* non-boundary elements of $f_{k}$. The matrix $B\in R^{(N-Q)\times N}$ is a selection matrix that forces elements of $f_{k}$ corresponding to the boundary to comply with the set boundary condition.

As a last step, we slightly regroup (3) to bring it into a more convenient form of

(5) $$\begin{array}{c} \underset{{\tilde{A}}_{k}}{\underset{︸}{\left(\frac{1}{\Delta T}I_{Q\times N}-\kappa \;L+v_{k}^{\;T}\;D\right)}}\;f_{k}-\underset{\tilde{C}}{\underset{︸}{\left(\frac{1}{\Delta T}\;I_{Q\times N}\right)}}\;f_{k-1}=I\;q. \end{array}$$

**Figure 1:**
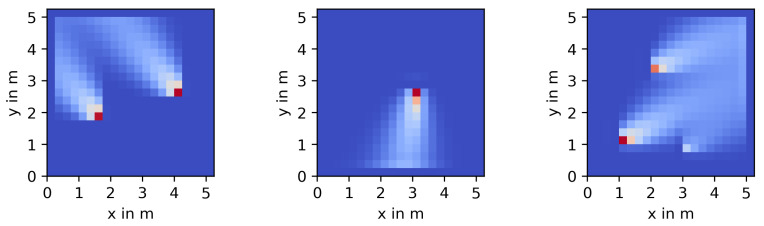
Three different snapshots of forward simulations of the process model.

Figure 1 shows three examples of how concentration fields $f_{k}$ for this process can look like, as rendered by our forward simulator for a resolution of N=21×21=441 discretization cells. The simulation setup will be introduced in more detail in Section 3. Shown are cases where q contains 2, 1, or 3 sources, respectively.

### 2.2. Measurements

To take concentration measurements, we utilize a swarm of *A* robots equipped with in situ gas concentration sensors. Each robot a=1,…,A, occupying a position $p_{a,t}$ within grid cell $x_{n}$, is exposed to the concentration value present in that grid cell, as concentrations are modelled to be constant throughout the space of a grid cell. More formally, we model the noisy measurement $z_{a,t}$ performed by the agent *a*, at a discrete time $t_{i}\in R_{+}$ within the time interval designated the index *k*, as

(6) $$\begin{array}{c} z_{a,t_{i}}=m^{\;T}\left(p_{a,t_{i}}\right)\;f_{k}+\xi_{a,t_{i}}, \end{array}$$

where $m\left(p_{a,t_{i}}\right)\in R^{N}$ is a selection vector that contains a 1 at index *n*, i.e., the index of the element corresponding to the location $p_{a,t_{i}}$ in the discretized representation of Ω, and that is 0 everywhere else. The perturbation $\xi_{a,t_{i}}$ is modeled as random, normally distributed noise with zero-mean and a variance of $\sigma_{z}^{2}$.

By collecting the measurements of all agents in a vector, we can rewrite (6) as

(7) $$z_{k}=m_{k}\;f_{k}+\xi ,$$

where $M_{k}={\left[m\left(p_{1,t_{1}}\right),m\left(p_{1,t_{2}}\right),\ldots ,m\left(p_{A,t_{1}}\right),\ldots \right]}^{\;T}$ and $\xi ={\left[\xi_{1,t_{1}},\xi_{1,t_{2}},\ldots ,\xi_{A,1},\ldots \right]}^{\;T}$.

This vector $z_{k}$ will collect all measurements that happen during the interval designated the time index *k*, taken by any agent $a\in \left[1,\ldots ,A\right]$. Since each agent may take multiple measurements during this duration, the length of $z_{k}$ may in fact be larger than *A*.

We are now ready to formulate a probabilistic framework that forms the basis for the proposed exploration algorithms.

### 2.3. Bayesian Estimation of Process Parameters

**Figure 2:**
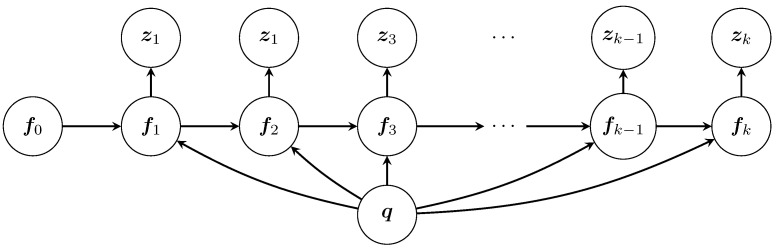
Graph representation of the underlying probabilistic structure of the Bayesian estimation.

Our ultimate objective is to find the source distribution represented by the vector q. This implcitly will require knowledge of the concentration distributions $f_{1},\ldots ,f_{k}$ based on the collected measurements $z_{1},\ldots ,z_{k}$. In other words, we are interested in computing the posterior $p(q,f_{k},\ldots ,f_{0}|z_{k},\ldots ,z_{1})$, which we can construct iteratively as follows. For this, please refer to Figure 2 for a graphical representation. We begin with k=1. In this case,

(8) $$\begin{array}{c} p\left(q,f_{1},f_{0}|z_{1}\right)\;\propto \;p\left(z_{1}|f_{1}\right)p\left(f_{1}|q,f_{0}\right)p\left(q\right)p\left(f_{0}\right), \end{array}$$

with initial priors p(q) and $p(f_{0})$. We will discuss those later in the text. For the next time step k=2 we can extend the posterior as

(9) $$\begin{array}{cc} p(q,f_{2},f_{1},f_{0}|z_{2},z_{1})\;\propto \; & p\left(z_{2}|f_{2}\right)p\left(f_{2}|q,f_{1},f_{0}\right)p\left(q,f_{1},f_{0}|z_{1}\right) \end{array}$$

(10) $$\begin{array}{cc} \;\;\;\;\;\;\;\;\propto \; & p\left(z_{2}|f_{2}\right)p\left(f_{2}|q,f_{1}\right)p\left(q,f_{1},f_{0}|z_{1}\right), \end{array}$$

where we use the fact that $f_{2}$ is conditionally independent of $f_{0}$ given q and $f_{1}$ and reused the posterior computed at the time step k=1 in (8). Let us point out that this conditional independence follows immediately from the PDE model (3). In general, this leads to the following update rule:

(11) $$\begin{array}{c} p\left(q,f_{k},\ldots ,f_{0}|z_{k},\ldots ,z_{1}\right)\;\propto \;p\left(z_{k}|f_{k}\right)p\left(f_{k}|q,f_{k-1}\right)p\left(q,f_{k-1},\ldots ,f_{0}|z_{k-1},\ldots ,z_{1}\right). \end{array}$$

Note that $p(f_{k}|q,f_{k-1})$ in essence encodes our process model according to (5). For a perfect model fit, i.e., when (5) holds exactly, we have $p\left(f_{k}|q,f_{k-1}\right)=\delta_{\tilde{f_{k}}}\left(R^{N}\right)$, where $\tilde{f_{k}}$ is the support of a Dirac delta function, such that ${\tilde{A}}_{k}{\tilde{f}}_{k}+{\tilde{C}}_{k}f_{k-1}=q$ (see (5)). We, however, relax (5) as well as the associated boundary condition (4) by allowing stochastic deviations from the equality. Specifically, we use a Gaussian distribution (see also [24]) to model deviations from the exact equalities such that

(12) $$\begin{array}{c} p\left(f_{k}|q,f_{k-1}\right)\;\propto \;\exp\left(-\frac{\lambda_{A}^{2}}{2}||{{\tilde{A}}_{k}{\tilde{f}}_{k}+\tilde{C}f_{k-1}-q||}_{2}^{2}-\frac{\lambda_{B}^{2}}{2}{\left||B\;f|\right|}_{2}^{2}\right). \end{array}$$

Here, the parameters $\lambda_{A}$ and $\lambda_{B}$ represent the “degree” of model relaxation: as they grow, the relaxed model approaches exact equality.

Further, as we see from (11), we need to define the likelihood of the measurements $p(z_{k}|f_{k})$. Based on (7), this can be formulated as

(13) $$\begin{array}{c} p\left(z_{k}|f_{k}\right)\;\propto \;\exp\left(-\frac{\lambda_{z}^{2}}{2}||{M_{k}\;f_{k}-z_{k}||}_{2}^{2}\right), \end{array}$$

where we define $\lambda_{z}^{2}=\sigma_{z}^{-2}$.

Let us now discuss the selection of prior distributions for q and $f_{0}$. We assume that the gas concentration at time k=0 is zero everywhere. Thus, we set

(14) $$\begin{array}{c} p\left(f_{0}\right)\;\propto \;\exp\left(-\frac{\tau_{f_{0}}}{2}{\left||f_{0}|\right|}_{2}^{2}\right) \end{array}$$

We will set $\tau_{f_{0}}$ to a high value that accounts for our “trust” in the initial condition $f_{0}=0$. For q, we similarly assume

(15) $$\begin{array}{c} p\left(q\right)\;\propto \;\exp\left(-\frac{\tau_{q}}{2}{\left||q|\right|}_{2}^{2}\right) \end{array}$$

with the parameter $\tau_{q}$ controlling the width of the prior and acting as regularization parameters. In fact, as we will see later, this form of the prior will act as a $\ell_{2}$ regularization of the source distribution estimate.

As can be seen from (11), the posterior is a multivariate Gaussian distribution that is growing in dimension with every new time step. To reduce the computational complexity, we cut off the history at k−1 by marginalizing over older concentration distributions $f_{k-2},\ldots ,f_{0}$, i.e.,

(16) $$\begin{array}{c} p\left(q,f_{k},f_{k-1}|z_{k},\ldots ,z_{1}\right)=\int \ldots \int p\left(q,f_{k},\ldots ,f_{0}|z_{k},\ldots ,z_{1}\right)df_{k-2},\ldots ,df_{0} \end{array}$$

This marginalised posterior can also be calculated in an iterative fashion with the following update rule:

(17) $$\begin{array}{c} p\left(q,f_{k},f_{k-1}|z_{k},\ldots ,z_{1}\right)\;\propto \;p\left(z_{k}|f_{k}\right)p\left(f_{k}|q,f_{k-1}\right)p\left(q,f_{k-1}|z_{k-1},\ldots ,z_{1}\right), \end{array}$$

with

(18) $$\begin{array}{c} p\left(q,f_{k-1}|z_{k-1},\ldots ,z_{1}\right)\;\propto \;\int p\left(q,f_{k-1},f_{k-2}|z_{k-1},\ldots ,z_{1}\right)df_{k-2} \end{array}$$

where $p(q,f_{k-1},f_{k-2}|z_{k-1},\ldots ,z_{1})$ was calculated in the previous time step k−1 according to (17). Note that (18) can be easily shown to be Gaussian, which follows from the gaussianity of the likelihood (13), relaxed model (12), and priors (14) and (15). As a consequence, it can be computed as

$$p\left(q,f_{k-1}|z_{k-1},\ldots ,z_{1}\right)=N\left(\left.\left(\begin{array}{c} f_{k-1}\\ q \end{array}\right)|\left(\begin{array}{c} {\hat{f}}_{k-1}\\ {\hat{q}}_{k-1} \end{array}\right),\right.\;{\hat{\Sigma }}_{k-1}\right).$$

Finally, making use of the fact that all factors on the right-hand side of (17) are normal, the marginalized posterior $p(q,f_{k},f_{k-1}|z_{k},\ldots ,z_{1})$ is also Gaussian. As such, after relatively simple algebraic manipulations we can express it as

(19) $$\begin{array}{cc} \; & \ln p\left(q,f_{k},f_{k-1}z_{k},\ldots ,z_{1}\right)\;\propto^{e}\;\\ \; & -\frac{1}{2}\;{∥\underset{p}{\underset{︸}{\left(\begin{array}{ccc} \lambda_{z}M_{k} & 0 & 0\\ \lambda_{A}{\tilde{A}}_{k} & \lambda_{A}\tilde{C} & -\lambda_{A}I\\ \lambda_{B}B & 0 & 0\\ 0 & \;\;{\hat{\Sigma }}_{k-1}^{-\frac{1}{2}} \end{array}\right)}}\underset{\theta }{\underset{︸}{\left(\begin{array}{c} f_{k}\\ f_{k-1}\\ q \end{array}\right)}}-\underset{\nu }{\underset{︸}{\left(\begin{array}{cccc} \lambda_{z}I & & 0 & 0\\ 0 & & 0 & 0\\ 0 & & 0 & 0\\ 0 & & \;\;{\hat{\Sigma }}_{k-1}^{-\frac{1}{2}} \end{array}\right)\left(\begin{array}{c} z_{k}\\ {\hat{f}}_{k-1}\\ {\hat{q}}_{k-1} \end{array}\right)}}∥}_{2}^{2} \end{array}$$

in matrix notation. Here, we also defined vectors ν, θ and a matrix p to simplify further notation. We note that in general, ν, θ, and a matrix p are all functions of the time index *k*. We make this dependency implicit to unclutter notation. Now, we can compute moments of $p\left(q,f_{k},f_{k-1}|z_{k},\ldots ,z_{1}\right)\equiv p\left(\theta |z_{k},\ldots ,z_{1}\right)$ by simply completing the square as follows:

(20) $$\begin{array}{cc} \ln p(\theta ,|z_{k},\ldots ,z_{1})\; & \propto^{e}\;-\frac{1}{2}\;{\left(p\theta -\nu \right)}^{\;T}\;\left(p\theta -\nu \right) \end{array}$$

(21) $$\begin{array}{cc} \; & \;\;\;\;\;\;\;\;\;=-\frac{1}{2}\;{\left(\theta -{\left(p^{T}p\right)}^{-1}p^{T}\nu \right)}^{\;T}\underset{\Sigma_{\theta }^{-1}}{\underset{︸}{\;p^{\;T}p\;}}\left(\theta -\underset{\mu }{\underset{︸}{{\left(p^{T}p\right)}^{-1}p^{T}\nu }}\right), \end{array}$$

where ${\left(p^{T}p\right)}^{-1}p^{T}$ is the pseudo-inverse of p, and $\Sigma_{\theta }={\left(p^{\;T}p\right)}^{-1}$ and $\mu ={\left(p^{T}p\right)}^{-1}p^{T}\nu$ are the covariance and mean of $p(\theta ,|z_{k},\ldots ,z_{1})$, respectively. Naturally, the mean μ readily provides the MAP estimate of the posterior source q as well as states $f_{k}$ and $f_{k-1}$.

Let us now inspect the covariance matrix $\Sigma_{\theta }$ in more detail. Using the structure of p we can show that

(22) $$\begin{array}{c} \Sigma_{\theta }={\left(p^{\;T}p\right)}^{-1}=\left(\begin{array}{ccc} \Sigma_{f_{k},f_{k}} & \Sigma_{f_{k},f_{k-1}} & \Sigma_{f_{k},q}\\ \Sigma_{f_{k-1},f_{k}} & \Sigma_{f_{k-1},f_{k-1}} & \Sigma_{f_{k-1},q}\\ \Sigma_{q,f_{k}} & \Sigma_{q,f_{k-1}} & \Sigma_{q,q} \end{array}\right). \end{array}$$

Clearly, evaluating $\Sigma_{\theta }$ is computationally quite expensive (the dimension of $\Sigma_{\theta }$ is (2N+Q)×(2N+Q), which can be substantial in practice), yet this is the price for computing the uncertainty estimates of individual cells and is required for the proposed exploration strategy.

For the exploration purposes we are more interested in the uncertainties related to $f_{k}$ and q. These can be obtained by “extracting” specific block matrices from $\Sigma_{\theta }$:

(23) $$\begin{array}{c} {\hat{\Sigma }}_{k}=\left(\begin{array}{cc} \Sigma_{f_{k},f_{k}} & \Sigma_{f_{k},q}\\ \Sigma_{q,f_{k}} & \Sigma_{q,q} \end{array}\right). \end{array}$$

Indeed, each of the *N* diagonal elements of $\Sigma_{f_{k},f_{k}}$ corresponds to one grid cell, thus giving us a certainty or, in the case of (23), the variance at a particular spatial location. These variances reveal our (un-)certainty about an estimation of the concentration value at a particular cell; we will refer to the aggregation of all these variances, $\sigma_{f_{k}}^{2}=\mathrm{diag}\left(\Sigma_{f_{k},f_{k}}\right)$, as the *uncertainty maps*. Specifically,

- *High variance* of a particular cell would indicate little information about the concentration at this particular location;
- *Low variance* would indicate high information content, and thus changes in this particular element will have a strong impact on the deviations of the cost function from the MAP optimum.

Likewise, the diagonal $\sigma_{q}^{2}=\mathrm{diag}\left(\Sigma_{q,q}\right)$ represents variances of the estimated source release rates at the corresponding *Q* locations. The objective of exploration will thus be to reduce this uncertainty, i.e., some measure of $\sigma_{f_{k}}^{2}$ and/or $\sigma_{q}^{2}$ by taking measurements in strategic locations.

**Figure 3:**
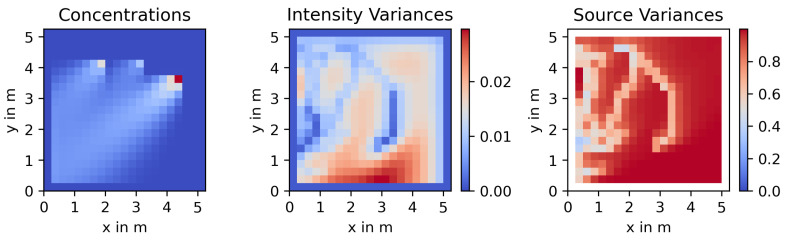
Representative intensity and source uncertainty maps for the process shown on the left.

Figure 3 shows a representative example of the uncertainty maps derived in this section. As before, the chosen N=441 yields a resolution of 21×21 grid cells. The process realization in question is being explored by two agents, and has three sources and a dominant wind vector pointing left and down. This will cause intensity variances ($\sigma_{f_{k}}^{2}$) to “pool” along the left and bottom edge of the map. Because of this, as we will introduce later on, our agents will start to roam into this area and take measurements there. Wherever rovers have been, both the intensity variances (middle) as well as the source variances ($\sigma_{q}^{2}$, right) will be reduced. Because of this, the past trajectories become visible, especially in the source variances. At the snapshot shown in this example, the uncertainty has been reduced on the left edge of the map already, so agents should next focus on the bottom edge, where intensity variances are highest (dark red area).

In the following sections we will discuss in more detail the exploration strategy with which we propose to solve this task, which exploits $\sigma_{f_{k}}^{2}$ to guide the robots towards more informative sampling locations.

### 2.4. Robot Model

**Figure 4:**
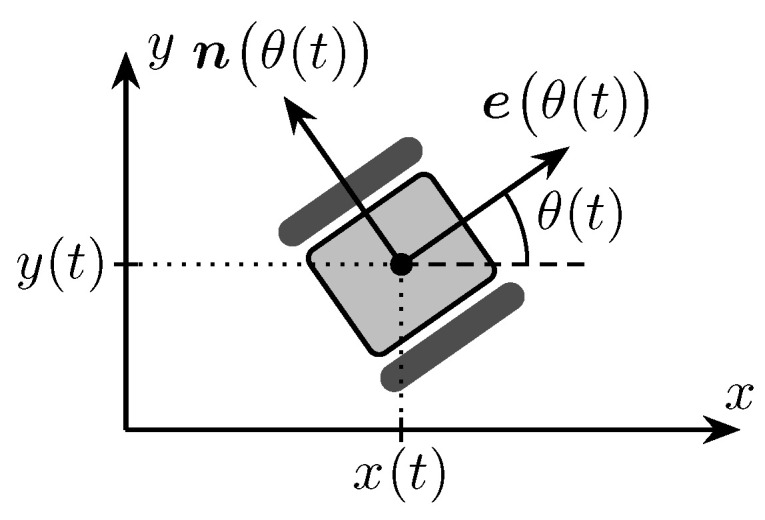
Robot Geometry (from [17]).

To explore the distribution of an unknown gas source, we deploy a swarm of mobile robots capable of measuring gas intensity at their respective positions. To implement the control law discussed in the following sections, we require a motion model for the robots. Therefore, we refer back to the model outlined in our previous work (cf. [17]), which we will restate here. We consider a swarm N{1,…,A} of *A* mobile track-driven robots. Thus, we use a simple *unicycle*-type motion model to represent each individual robot a∈N. The state of a robot is fully described by its 2D position, $p_{a}\left(t\right)={\left(\begin{array}{cc} x_{a}\left(t\right) & y_{a}\left(t\right) \end{array}\right)}^{\;T}\in R^{2}$, and the heading angle $\theta_{a}\left(t\right)\in \left[0;2\pi \right)$. The heading can equivalently be expressed in terms of the forward and normal orientation vectors, $e_{a}\left(\theta_{a}\left(t\right)\right)$ and $n_{a}\left(\theta_{a}\left(t\right)\right)$, as depicted in Figure 4. The dynamics of robot *a* are described using the state-space model

(24) $$\begin{array}{c} R_{a}:\left\{\begin{array}{c} \begin{array}{cc} {\dot{p}}_{a}\left(t\right) & =e_{a}\left(\theta_{a}\left(t\right)\right)\;u_{v,a}\left(t\right)\\ {\dot{\theta }}_{a}\left(t\right) & =u_{\omega ,a}\left(t\right), \end{array} \end{array}\right. \end{array}$$

that captures its non-holonomic constraints. Here, $u_{v,a}\left(t\right)$ represents the scalar control input for linear velocity, which corresponds to forward/backward motion, while $u_{\omega ,a}\left(t\right)$ denotes the control input for angular velocity, representing the turn rate. The computation of these control inputs is the responsibility of the control laws, derived in the following section.

### 2.5. Potential Field Control

In this section, our aim is to develop a control scheme for the robots with the objective to collect measurements as efficiently as possible. Essentially, the controller should guide the robots to locations with high values in the uncertainty map. To achieve this, we employ an Artificial Potential Field Control approach.

Artificial Potential Field Control is an approach from control theory that lends itself well to the control of mobile multi-robot systems. Conceptually, it operates by constructing a potential function across the spatial environment. This function assigns a *potential* value to each point within this space. To navigate, each agent only needs to evaluate the gradient of said potential function at its location. The field of these gradients is the so-called *potential field*. By following the negative gradients of the potential, agents are automatically steered toward local minima in the potential function. Consequently, the primary task of the control engineer centers around designing an appropriate potential function. In this context, regions of interest or goal positions are intended to be low points in the potential function, which attract the robots. Regions that should be avoided, such as for collision avoidance, should be elevated, creating peaks that repel the robots.

The potential function itself needs to be a continuously differentiable function P:Ω→R over the area of interest, Ω. Its spatial gradient $\frac{dP}{dx}$ is the *potential field*. This vector field can be interpreted as virtual forces that act upon the robots.

The potential function *P* can be conveniently constructed as the sum of several contributing components. In our case, we take into account $P=P_{\mathrm{rep}}+P_{\mathrm{attr}}$, that is a repulsive component $P_{\mathrm{rep}}$ which pushes rovers apart when they come into close proximity and ensures collision avoidance, and an attractive component $P_{\mathrm{attr}}$ that guides agents towards informative locations. Additional components could be taken into account in a straightforward manner. Arbitrarily shaped obstacles [25] are an example known in the literature, as well as communication constraints between robots, among others. As these are well established in the field, they are not incorporated here, although their effect on the performance of the exploration scheme pose an interesting research question for future work.

In the remainder of this section, we will discuss the design of the components of the potential function *P*. It is important to note that these, as well as the control laws derived from them in the end, are identical to the versions presented in our previous work (cf. [17]), and are stated here again for the sake of completeness.

#### 2.5.1. Repulsive Potential

We design the repulsive potential $P_{\mathrm{rep}}\left(x\right)$, x∈Ω, as the sum of individual repulsive potentials $\varphi_{\mathrm{rep}}\left(x,p_{a}\left(t\right)\right)$ around the position of each agent *a*:

(25) $$\begin{array}{cc} \varphi_{\mathrm{rep}}\left(x,p_{a}\left(t\right)\right)\; & =\left\{\begin{array}{cc} \frac{k_{R}}{2}\;{\left(∥x-p_{a}\left(t\right)∥-\delta \right)}^{2},\; & ∥x-p_{a}\left(t\right)∥\leq \delta \\ 0,\; & \mathrm{otherwise}, \end{array}\right. \end{array}$$

(26) $$\begin{array}{cc} \mathrm{such}\;\mathrm{that}\;P_{\mathrm{rep}}\left(x\right)\; & =\sum_{a\in N}\left(\frac{1}{2}\sum_{b\in N\setminus a}\varphi_{\mathrm{rep}}\left(p_{b}\left(t\right),p_{a}\left(t\right)\right)\right). \end{array}$$

The fact that $\varphi_{\mathrm{rep}}\left(x,p_{a}\left(t\right)\right)$ vanishes for distances from robot *a* larger than δ implies that only the local neighborhood of any robot is relevant for its collision avoidance. With the derivative of each individual $\varphi_{\mathrm{rep}}\left(x,p_{a}\left(t\right)\right)$ (see Equation (27)), the potential field takes the form of Equation (28):

(27) $$\begin{array}{cc} \; & \frac{𝜕\varphi_{\mathrm{rep}}\left(x,p_{a}\left(t\right)\right)}{𝜕x}=\left\{\begin{array}{cc} k_{R}\;\left(1-\frac{\delta }{∥x-p_{a}\left(t\right)∥}\right)\;{\left(x-p_{a}\left(t\right)\right)}^{\;T},\; & ∥x-p_{a}\left(t\right)∥\leq \delta \\ 0,\; & \mathrm{otherwise}, \end{array}\right. \end{array}$$

(28) $$\begin{array}{cc} \; & \mathrm{which}\;\mathrm{leads}\;\mathrm{to}\;{\left.\frac{𝜕P_{\mathrm{rep}}\left(x\right)}{𝜕x}\right|}_{x=p_{a}\left(t\right)}=\sum_{b\in N\setminus a}\left({\left.\frac{𝜕\varphi_{\mathrm{rep}}\left(x,p_{a}\left(t\right)\right)}{𝜕x}\right|}_{x=p_{b}\left(t\right)}\right). \end{array}$$

The quadratic term in (25) creates a steeply increasing potential where robots get close to each other. This leads to a linearly increasing repulsion “force” the closer two robots get, which we can scale appropriately using the parameter $k_{R}$.

#### 2.5.2. Attractive Potential

We will next discuss the design of the attractive part of the potential function, $P_{\mathrm{attr}}\left(x\right)$, based on the computed uncertainty map $\sigma_{f_{k}}^{2}$ (cf. Section 2.3). Our objective is to make agents gravitate towards regions of high uncertainty. We assume that a new measurement at such a location with high uncertainty is more informative than measurements at a location with already low uncertainty. The reason for basing this potential function on $\sigma_{f_{k}}^{2}$ over $\sigma_{q}^{2}$ is motivated by the fact that measurements also sample, and thus directly reduce the uncertainty of, $f_{k}$. First, we normalize $\sigma_{f_{k}}^{2}$ to the range $\left[0;1\right]$, as the absolute scale of uncertainty is not of interest. To obtain from this a continuously differentiable potential function, we create an interpolation using a bivariate cubic spline,

(29) $$\varphi \left(x\right)=\mathrm{CubicSpline}\left(\frac{\sigma_{f_{k}}^{2}}{\max_{i,j}\sigma_{f_{k}}^{2}}\right),\;s.t.\;{∥\varphi -\frac{\sigma_{f_{k}}^{2}}{\max_{i,j}\sigma_{f_{k}}^{2}}∥}_{2}^{2}\leq N\sigma_{s}^{2},$$

where φ is a vector aggregating elements $\varphi \left(x_{i,j}\right),x_{i,j}\in \Omega$, in the same manner as $f_{k}$ (see Equation (3)), and *N* is the number of grid cells, as before. The additional constraint allows to smooth out small local extrema in φ(x); we choose $\sigma_{s}=0.05$. Being a polynomial representation makes φ(x) trivial to differentiate. Notice that it is a function of space, as $\sigma_{f_{k}}^{2}$ implicitly depends on space as well.

Differentiating φ(x) directly is not desirable, however, as there are no bounds to its steepness. Instead, we modify the vector field one last time; specifically:

(30) $$\frac{dP_{\mathrm{attr}}\left(x\right)}{dx}=\frac{g(x)}{\alpha +∥g(x)∥},\;\mathrm{where}\;g\left(x\right)=-\nabla \varphi \left(x\right).$$

This vector field exposes robots to a bounded virtual force, until they come close to an extremum, where they will stop and remain until the potential function updates. Note that Equation (30) is akin to a sigmoid function with a saturation effect that ensures control input limits are not violated, and can be tuned with a parameter α.

It should be noted that $\frac{dP_{\mathrm{attr}}\left(x\right)}{dx}$ specifies the attractive field acting on the robots. It is possible, however, that due to the saturation introduced in Equation (30) this field can no longer be expressed as the gradient of a scalar potential function $P_{\mathrm{attr}}\left(x\right)$; in other words, it may no longer be conservative. Yet, for our purposes this technicality is irrelevant, as explicit $P_{\mathrm{attr}}\left(x\right)$ is not needed for navigation.

We would like to point out again that the attractive part of the potential function, along with all modifications made to it, are applied in this work just like they have been presented in our previous publication [17], as mentioned before, to which this paper is an extension. We do this with the aim of evaluating how this method of constructing a potential field fares with the new and improved process description using the advection–diffusion equations (cf. Equation (1)).

#### 2.5.3. Control Laws

To descend along the gradient of the potential function *P*, the robots need to align themselves with the potential field. As in our previous work [17]), we use

(31) $$\begin{array}{cc} u_{\omega ,a}\left(t\right)\; & =k_{\omega }\;\arctan\;\left(\frac{n_{a}{\left(\theta_{a}\left(t\right)\right)}^{\;T}\;\frac{𝜕P}{𝜕p_{a}}}{e_{a}{\left(\theta_{a}\left(t\right)\right)}^{\;T}\;\frac{𝜕P}{𝜕p_{a}}}\right) \end{array}$$

(32) $$\begin{array}{cc} u_{v,a}\left(t\right)\; & =-k_{v}\;{\frac{𝜕P}{𝜕p_{a}}}^{\;T}\;e_{a}\left(\theta_{a}\left(t\right)\right), \end{array}$$

as control laws for the angular and forward velocity of robot *a*. The controller gains $k_{\omega }>0$ and $k_{v}>0$ are again set heuristically.

The role of the turn rate control law $u_{\omega ,a}\left(t\right)$ is to turn the robot until it is aligned with the gradient’s direction, with either its front or its rear facing down the potential hill. Since the robot can drive both forwards or backwards, and it is thus irrelevant which side is turned in the desired direction, the control law is chosen such that it takes the angle that is closest.

The control law $u_{v,a}\left(t\right)$ adjusts the velocity dependent on the robots angle relative to the gradient of *P*. The vector product between the gradient and the robot’s unit forward vector $e_{a}\left(\theta_{a}\left(t\right)\right)$ has the following effect: As the vector product will be zero when both vectors are orthogonal, i.e., when the robot is turned orthogonal to the potential field, the robot’s command linear velocity will become zero in this case; however, the robot’s angular velocity will make the robot turn. The maximum command velocity at a particular location can be reached if the two vectors are perfectly in line with each other, that is, when the robot is facing straight “uphill” or “downhill” of the potential function. A more detailed discussion can be found in [18].

#### 2.5.4. Measurement Acquisition

Based on potential field navigation, robots are guided by the uncertainty map derived from the gas dispersion model to locations of high uncertainty. As robots continue to follow the local gradient in informativeness upwards, each subsequent point can be considered more informative than the previous one. Due to this effect, it is advantageous to collect measurements “along the way”, not only when reaching a local minimum in the uncertainty map, as was performed in our previous work [17]. Furthermore, in contrast to the case of static diffusion [17], the time-varying nature of the process means that only recent measurements provide helpful information for estimating the source distribution. In simple terms, the value and influence of older measurements fade out over time and are gradually forgotten. Therefore, taking more measurements across the area of interest in the current time step is advisable to obtain more recent information.

For these reasons, in our case, the robots sample the gas intensity at a constant rate of 10Hz throughout their trajectory. This is an important deviation from our approach in the previously presented conference paper [17], where we chose to take measurements only when we reached a peak of the potential function. While these peak points indeed promise to yield the maximum amount of information within their local neighborhood, continuous sampling allows us to obtain even more information simply by measuring more frequently. Additionally, we sample a larger subset of spatial locations in the same amount of time, which is beneficial for the reasons outlined above. However, it is important to note that this approach assumes that the robots are equipped with gas sensors capable of providing a fast response time of $\frac{1}{10}$ s (e.g., a photoionization detector).

For our simulation, we forward simulate the gas dispersion model (3) with an update rate of 1 Hz. This results in a gas intensity map $f_{k}$ for each of these time steps *k*. To simulate the gas sensor, we select the element of $f_{k}$ corresponding to the robot’s location and disturb the value by random Gaussian noise, as explained in Section 2.2.

## 3. Simulations

In this chapter, we will evaluate the presented approach in randomized simulation trials. We will test the exploration strategy presented thus far, and compare it against two benchmark strategies. All will be evaluated under static and fluctuating wind conditions, and judged using the performance metrics we will introduce in this section. For all cases, we will vary the swarm size and look at the influence this has on performance. The agent’s starting poses, wind direction, and source distribution are always randomized. A detailed analysis will follow in the discussion in Section 4. But first, we need to assign values to some remaining simulation parameters introduced in Section 2.

### 3.1. Simulation Parameters

The physical dimensions in our simulations are set in accordance to a typical, robotics laboratory environment. For discretization of the exploration domain, we choose a grid size of 0.25 m. This value can be considered as on the order of the physical dimensions of the robotic platforms. Further, it is small enough to capture a gas dispersion process in an indoor environment with sufficient resolution. With a grid of size 21 × 21, we end up with a total size of the exploration area of 5.25 × 5.25 m^2^. These are the same dimensions that were analyzed in our previous paper (cf. [17]).

For the control laws, we need to choose the controller gains. We set the velocity gain $k_{v}=0.3\;\frac{m}{s}$ to control the maximum cruising speed of the robots. The value of 0.3 $\frac{m}{s}$ is reasonable and achievable with typical hardware. It has to be remarked that the speed is an important factor. Reducing the speed means that robots have more time to collect information along their way. The turnrate gain is set as $k_{\omega }=1.5\;\frac{\mathrm{rad}}{s}$. It allows the rovers to traverse the potential field in a smooth manner, and has been chosen heuristically. The next control parameter is the separation constant in the repulsive potential field, which we set to δ=1.0 m. Once robots come within this distance from one another, they will begin to experience a repulsive force, pushing them apart to avoid collisions. The associated repulsion gain in the potential field is set as $k_{R}=0.2$. The overall potential field is smoothed using a cubic spline interpolation. To achieve smoothing, aside from pure interpolation, some error is allowed for at each support point. The amount of error is controlled by the parameter α=0.05.

As a last process parameter, the variance for the measurement noise, $\sigma_{z}^{2}$, is chosen in relation to the intensity values we can expect to measure using a typical gas sensor in practice. Thus, we settled on a value of $\sigma_{z}=0.2$. Keep in mind that this value is coupled to the process matrix $\tilde{A}$ by the equality $\lambda_{z}^{2}=\sigma_{z}^{-2}$ (cf. Equation (13)). While this fixes the value of $\lambda_{z}^{2}$, we set $\lambda_{B}^{2}=1000$ to signify a very strong confidence in the boundary condition and $\lambda_{A}^{2}=3$ to give some weight to the process model (cf. Equation (12)).

### 3.2. Performance Metrics

As we consider a dynamic process in this paper, comparing intensity reconstruction to its ground truth is less meaningful. Since the primary goal is anyhow localizing gas sources, comparing the reconstructed gas source distribution to the static ground truth is more meaningful. Assigning a good metric to the quality of a reconstructed source distribution can be quite elusive, because the reconstruction can deviate from the ground truth several different ways. The following errors can manifest in the reconstruction:

- Wrong number of sources (too many, too few);
- Sources at the wrong location;
- Wrong source release rates (intensities).

The last one is, however, less relevant if we are interested in source localization only.

**Figure 5:**
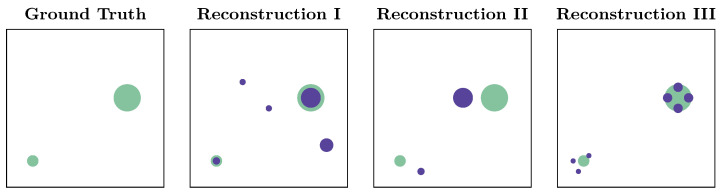
Three possible erroneous reconstructions, with ground truth always shown in green; size indicates source strength. Which reconstruction is worse?

For illustration, consider the following ground truth and the three possible reconstructions presented next to it (cf. Figure 5). The first reconstruction has the wrong number of sources compared to the ground truth but contains the true source locations with matching intensities. The second correctly identifies the number of sources but with slight location errors, and the third detects smaller sources roughly in the correct locations but fails to reconstruct individual sources accurately. It is quite challenging to determine which is better or worse, as it depends on the application and the trade-off between missing or having false detections. We see this ambiguity as a still open question in GSL when searching for multiple sources.

For this article, to evaluate the reconstruction performance we use the following metrics. Here, the absolute strength of the sources is less important, compared to the number and the locations of sources. As we mainly view accident and disaster response scenarios as our application case, it is most important to *find* source candidates, and find them quickly. Their precise strength, as well as their precise location, are less important than getting to know the approximate locations, quickly.

To score the source reconstructions, we extract their local maxima. We consider a candidate point p∈Ω to be a maximum and, therefore, as a source location, if it satisfies both of the following conditions:

- The reconstructed value $\hat{q}\left(p\right)$ is the largest of any of the $\hat{q}\left(x\right)$ in the local surroundings S(p) of p, i.e., $\forall x\in S\left(p\right):\hat{q}\left(p\right)\geq \hat{q}\left(x\right)$.
- $\hat{q}\left(p\right)$ is at least 5 times larger than the smallest value of $\hat{q}\left(x\right)$ within S(p),
  i.e., $\hat{q}\left(p\right)\geq 5\;{\hat{q}}_{\min}$ for ${\hat{q}}_{\min}\min_{x\;\in \;S(p)}\hat{q}\left(x\right)$.

Points that satisfy these conditions we treat as the locations of the sources. For S(p), we chose a neighborhood with a radius of three grid cells, i.e., ${\left||x-p|\right|}_{2}\;\leq \;R$ with R=3·0.25 m. Now we can compare the set of source location in the reconstructed source distribution with the ground truth source distribution. To this end, we define five criteria/metrics:

1. Our first performance metric is the time step when the two sets are exactly identical for the first time. This time we denote as $T_{\mathrm{first}}^{\equiv }$. This metric can be calculated for each simulation run, and we can evaluate its statistics when running multiple simulations.
2. As a second metric, we consider the time step when the reconstruction is “nearly exact” for the first time compared to the ground truth. We define “nearly exact” to mean any source distribution that (I) has the correct number of source candidates, and (II) all source candidates are very close to their correct location, i.e., the average euclidean distance between p and its true position is less than 2 grid cells. The first time of the “nearly exact” reconstruction we denote as $T_{\mathrm{first}}^{\approx }$.
3. Our third metric takes into account that the reconstruction may hit upon the correct solution early on, then diverge from it temporarily, before it finally settles back into the correct distribution. So, we can define a time when the reconstruction is “wrong” for the last time. The time when the set of estimated source is last not exactly the ground truth is denoted as $T_{\mathrm{last}}^{\neg \equiv }$.
4. Similar to $T_{\mathrm{last}}^{\neg \equiv }$, we can also define the last time when the reconstruction is not “nearly exact”, where “nearly exact” is defined as in point two. This time we denote as $T_{\mathrm{last}}^{\neg \approx }$. In general, the following inequalities hold:
  (33) $$\begin{array}{c} T_{\mathrm{first}}^{\approx }\leq T_{\mathrm{first}}^{\equiv }, \end{array}$$
  (34) $$\begin{array}{c} T_{\mathrm{last}}^{\neg \approx }\leq T_{\mathrm{last}}^{\neg \equiv }. \end{array}$$
5. Last, we will have a look at the total source uncertainty, $\mathrm{tr}(\sigma_{q}^{2})$, over time. This metric expresses the sum of source uncertainties at all locations, giving us a measure of the total amount of remaining uncertainty in the system. In contrast to the other metrics, this can be calculated without knowing the true source distribution, even online during an experiment.

### 3.3. Source Distributions

The source distributions for our simulations are drawn randomly. We have made the following assumptions on the source distribution: We are interested in sparse distributions, consisting of a few, isolated sources. To this end, we first draw a uniformly distributed number between one and four reflecting the number of sources. Next, we randomly pick locations in the environment for these sources. To account for sparse, spread-out sources, and the Dirichlet boundary condition, we place additional constraints on the source spatial locations. The source locations have to fulfill the following conditions:

1. Sources must be at least 10% away from the edges.
2. Sources must be at least 20% away from each other.

The percentages are to be understood as relative to the edge lengths of the exploration area Ω, i.e., 5.25 m in our case. For each source location we determine the appropriate element in our source distribution vector q. The corresponding element is set to a random value between 50 $\frac{1}{s}$ and 500 $\frac{1}{s}$ for forward simulation of the gas dispersion process.

### 3.4. Wind Models

In our simulations, we want to evaluate our approach against two different wind models. As introduced in Section 2, the wind vector v(t) is assumed to be uniform over the entire exploration area.

As a first model, we assume a wind vector that is constant in time during the exploration mission. The direction of the vector is drawn randomly. For this, we draw an angle from a uniform distribution on the interval φ∈[0,2π) and set wind strength $\bar{v}$ to 0.3 $\frac{m}{s}$. This gives us

(35) $$\begin{array}{c} v\left(t\right)=\bar{v}\;{\left(\begin{array}{cc} \cos(\varphi ) & \sin(\varphi ) \end{array}\right)}^{\;T}. \end{array}$$

This simple wind model gives a nice baseline for the comparison of simulation runs. We will refer to this model as the “static wind” case.

**Figure 6:**
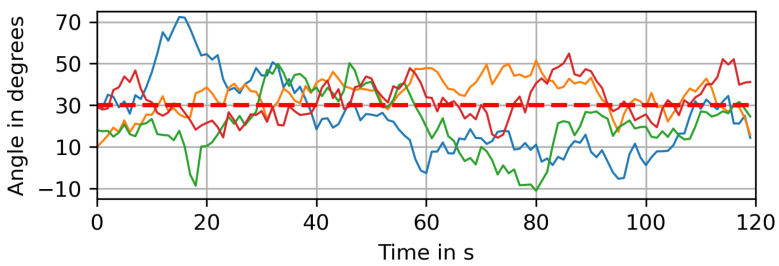
Several realizations of our fluctuating wind model over 2 min, with a dominant wind direction of $\varphi_{\mu }=30{}^{\circ }$ indicated as a red dashed line.

**Figure 7:**
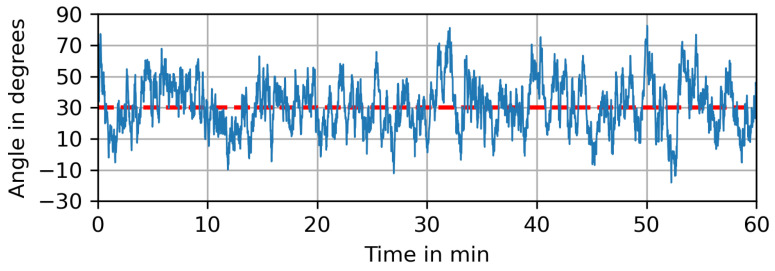
One realization of the fluctuating wind direction plotted over 60 min., with a dominant wind direction of $\varphi_{\mu }=30{}^{\circ }$ indicated as a red dashed line.

The second wind model we investigate aims to mimic realistic wind conditions that we would expect in outdoor experiments. In these scenarios, the wind shows a dominant wind direction that is static throughout a long period, but with strong fluctuations in momentary wind direction around the mean value on the timescale of several seconds. For a simple model of this behavior, we turn to an *Ornstein–Uhlenbeck process*. Essentially, this stochastic process constrains a random walk to a mean direction with a relaxation force, giving us a low pass filtered white noise [26]. The wind strength $\bar{v}$ is set to the same value as in the static wind case. This fluctuating wind model is defined as

(36) $$\begin{array}{cc} v_{k+1} & =\bar{v}\;{\left(\begin{array}{cc} \cos(\varphi_{k+1}) & \sin(\varphi_{k+1}) \end{array}\right)}^{\;T}, \end{array}$$

(37) $$\begin{array}{cc} \mathrm{with}\;\varphi_{k+1} & =\varphi_{k}+\gamma \;\left(\varphi_{\mu }-\varphi_{k}\right)+\eta_{k+1}, \end{array}$$

where $\eta_{k+1}$ is a noise term drawn from a normal distribution of $N\left(0,\sigma_{\varphi }^{2}\right)$ and $\varphi_{\mu }$ is the dominant wind direction, i.e., the mean value of this noise process. The parameter γ is the relaxation force, driving the process back to its mean value of $\varphi_{\mu }$. For the free parameters, we choose γ=0.05 and $\sigma_{\varphi }=5{}^{\circ }$. Figure 6 shows the time series of the wind direction for multiple realizations of the random process on a short time scale, while Figure 7 depicts one such realization over a longer period of time. In both cases the dominant wind direction is $\varphi_{\mu }=30{}^{\circ }$ and indicated as a red dashed line.

### 3.5. Benchmarks

Our approach shall be compared against two trivial exploration strategies as a means of a benchmark. This comparison shall yield a quantitative measure of how the proposed approach compares performance-wise against an approach without an information-seeking component.

**Figure 8:**
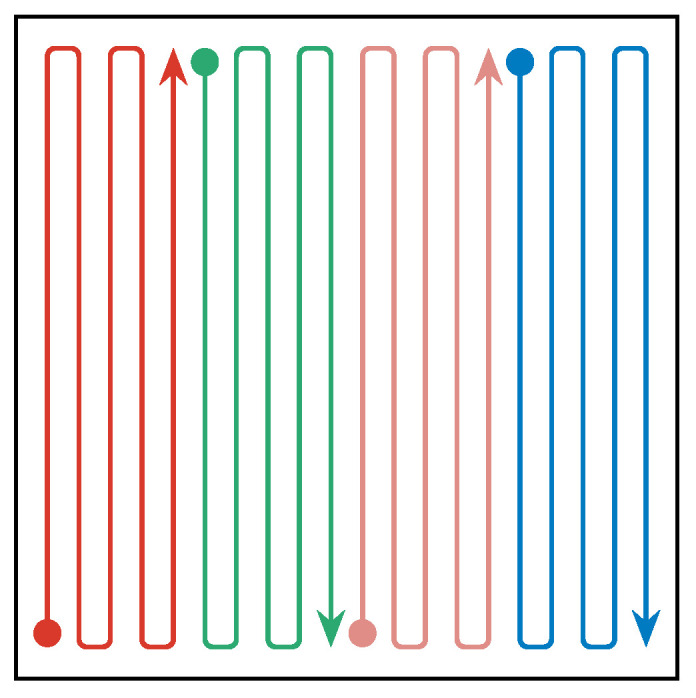
Schematic drawing of meander trajectories for a swarm of four agents.

The first benchmark strategy is a meandering trajectory. All swarm agents systematically sample the exploration area to achieve a full coverage of the area. Figure 8 shows an example of how a rectangular exploration area is split among four agents. The agents start at a random location as in all the other simulations. They first head to their starting positions, indicated as circles, and then follow trajectories in patterns as shown in the figure. These trajectories lead them trough all cells in the discretization grid. Once they reach the end of their path, they start over at the beginning.

The second benchmark strategy is an exploration scheme where agents essentially perform a random walk trough the exploration area Ω. Each agent randomly picks a movement direction, and follows it for 10 s. To keep all agents within the exploration area, agents are made to bounce off the edges of the exploration area, akin to light reflecting off a flat surface. To keep the comparison fair, the agents also need to avoid collisions among each other. In this way, we can study the impact that the number of robots has on the performance while also keeping the effect that the environment eventually becomes overcrowded with agents. To this end, we utilize the repulsive potential fields for collision avoidance.

## 4. Results and Discussion

### 4.1. Evaluation of Swarm Size

In a first step, we would like to analyze the effect of the number of robots on the performance of the reconstruction. We randomly choose 200 simulation environments consisting of a randomly drawn wind direction and a random source distribution in accordance with Section 3.3. We here employ the static wind model from Section 3.4 first. For every environment, we execute a simulation run of 10 min for a different swarm size A∈[1,2,3,5,10], i.e., the number of robots. To evaluate the effect of swarm size on performance, we first look at $T_{\mathrm{first}}^{\approx }$ and $T_{\mathrm{first}}^{\equiv }$. Based on the 200 simulation runs we generate a boxplot of these metrics. When a simulation does not reach the particular criterion within the 10 min, the corresponding time *T* is capped at 600 s.

**Figure 9:**
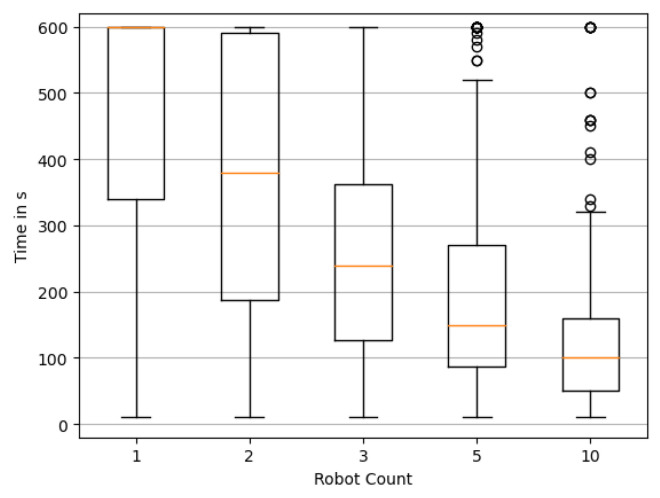
Distribution of the time required to reconstruct the source locations exactly. Box plots show quartile boundaries, and circles indicate outliers.

**Figure 10:**
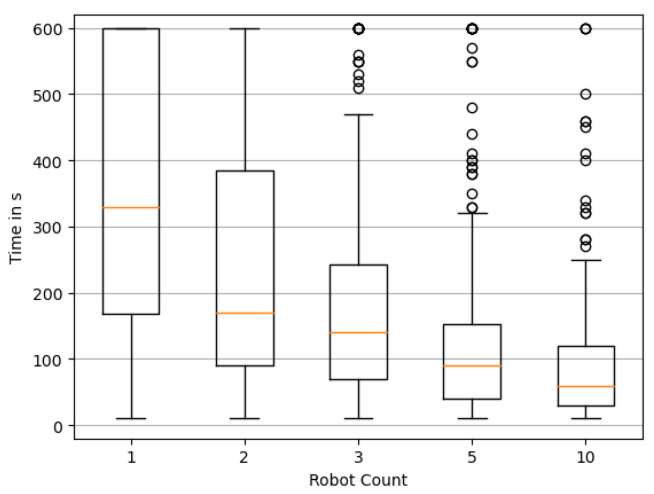
Distribution of the time required to reconstruct the source locations nearly exactly. Box plots show quartile boundaries, and circles indicate outliers.

Figure 9 shows $T_{\mathrm{first}}^{\equiv }$ for different numbers of robots. It is clearly observable that exploration benefits heavily from an increasing number of swarm agents. For a single agent it is hard to find the exact solution within the 10 min time frame, although it will sometimes happen by chance. The time to first reach an exact reconstruction drops rapidly with an increasing number of agents. Along with it, the variability in performance drops, visible in considerably smaller quartiles around the median. This means the performance gets more reliable and less dependent on chance. $T_{\mathrm{first}}^{\approx }$ shown in Figure 10 paints a similar picture. As apparent from the significantly reduced median times, a “nearly exact” solution can be found quite quickly by lager swarms, and of course as expected, more quickly than an exact reconstruction.

**Figure 11:**
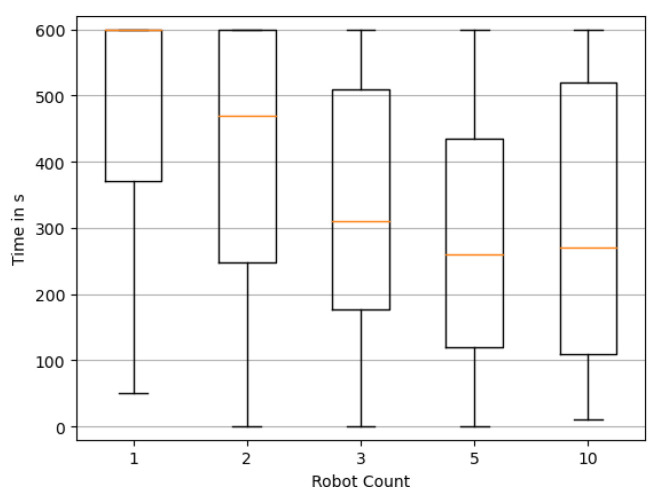
Distribution of the time required until the last reconstruction of source locations is not exactly correct. Box plots show quartile boundaries.

**Figure 12:**
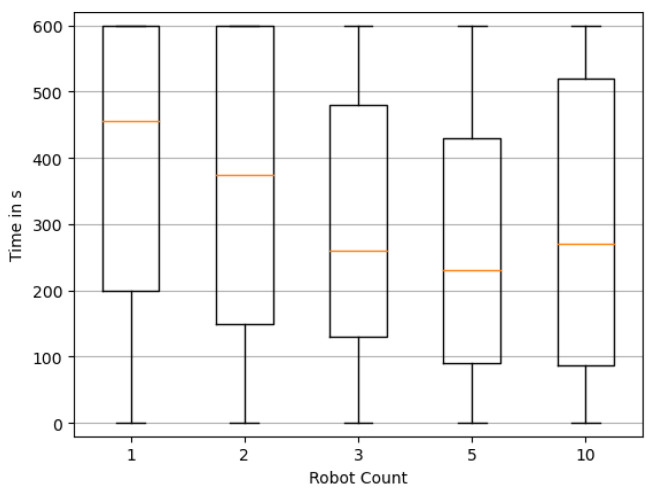
Distribution of the time required until the last reconstruction of source locations is not nearly exact. Box plots show quartile boundaries.

Figure 11 and Figure 12 confirm this trend for the duration until the reconstruction stays correct, or nearly correct. It is, however, apparent that for all cases, there also seems to be a trend of reconstructions deviating from the once correct solution again. Further research is needed to address how to detect when a correct solution has been achieved, and secondly, maintaining the reconstruction in such cases. While this phenomenon may pose a significant challenge, practical applications may in fact benefit from the assumption that true source candidates have previously been identified, enabling operators to act accordingly on this information already.

**Figure 13:**
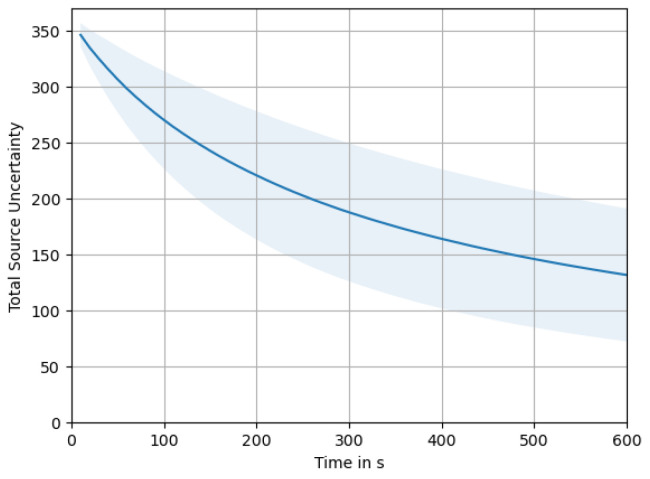
Total source uncertainty over time, averaged over all swarm sizes. Shaded area indicates the 1σ interval.

**Figure 14:**
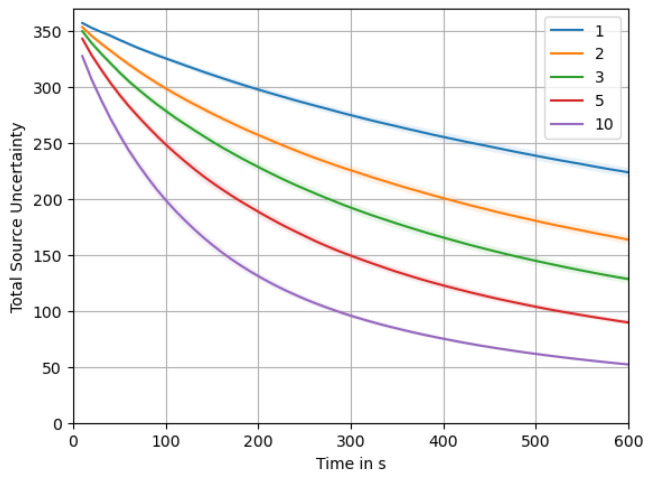
Total source uncertainty over time, averages broken down for different swarm sizes.

Figure 13 depicts the total source uncertainty, $\mathrm{tr}(\sigma_{q}^{2})$, over time averaged over all 5×200 simulations. As we can observe, $\mathrm{tr}(\sigma_{q}^{2})$ starts around an initial value of Q=361, influenced by our initialization based on the prior for the source distribution p(q). Over time, we see the uncertainty monotonically decrease as more and more measurements are taken. Within the chosen time frame of 10 min, the uncertainty does not reach a lower bound, but we would expect that it continues dropping asymptotically ad infinitum. Being a sum of variances, it will of course not drop below zero. The shaded area around the plot of the mean total source uncertainty shows a 1σ interval of the distribution over all simulation runs. The high variation indicated by the 1σ interval occurs due to the different swarm sizes. This becomes clear from a more detailed look at Figure 14, where the uncertainty is plotted for each swarm size. There we can also clearly see how larger swarms are able to reduce total uncertainty faster.

**Figure A1:**
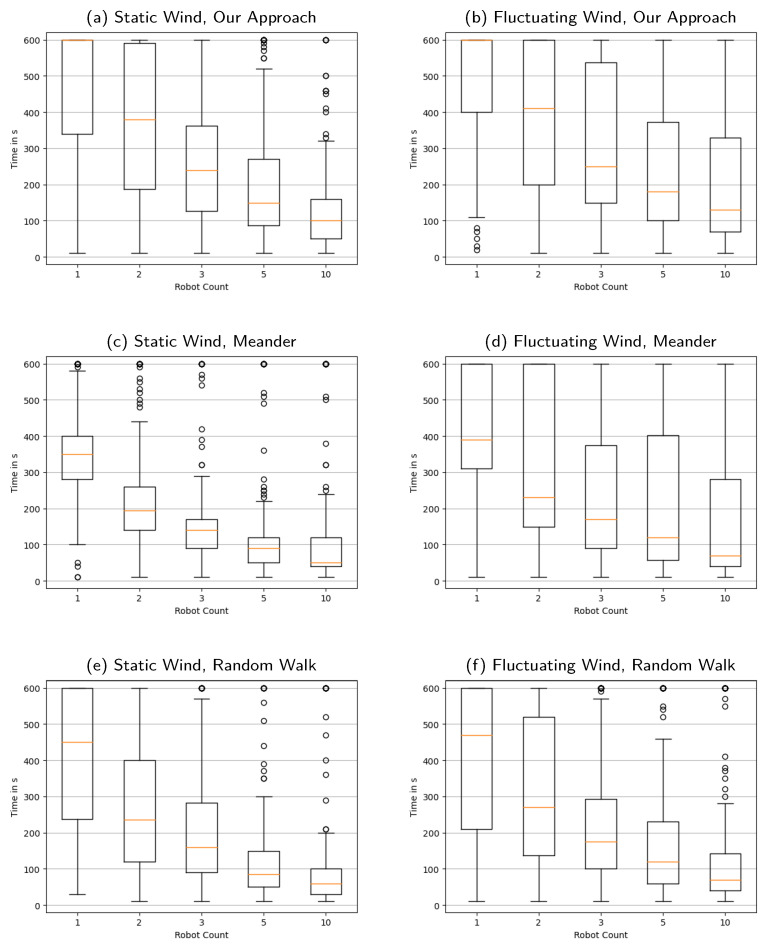
A side-by-side overview of $T_{\mathrm{first}}^{\equiv }$, i.e., the distributions of the time required to reconstruct the source locations *exactly*. Wind is static in the left column and fluctuating in the right. The first row presents our approach, the second is the meander benchmark, the third the random walk benchmark. Box plots show quartile boundaries, and circles indicate outliers.

**Figure A2:**
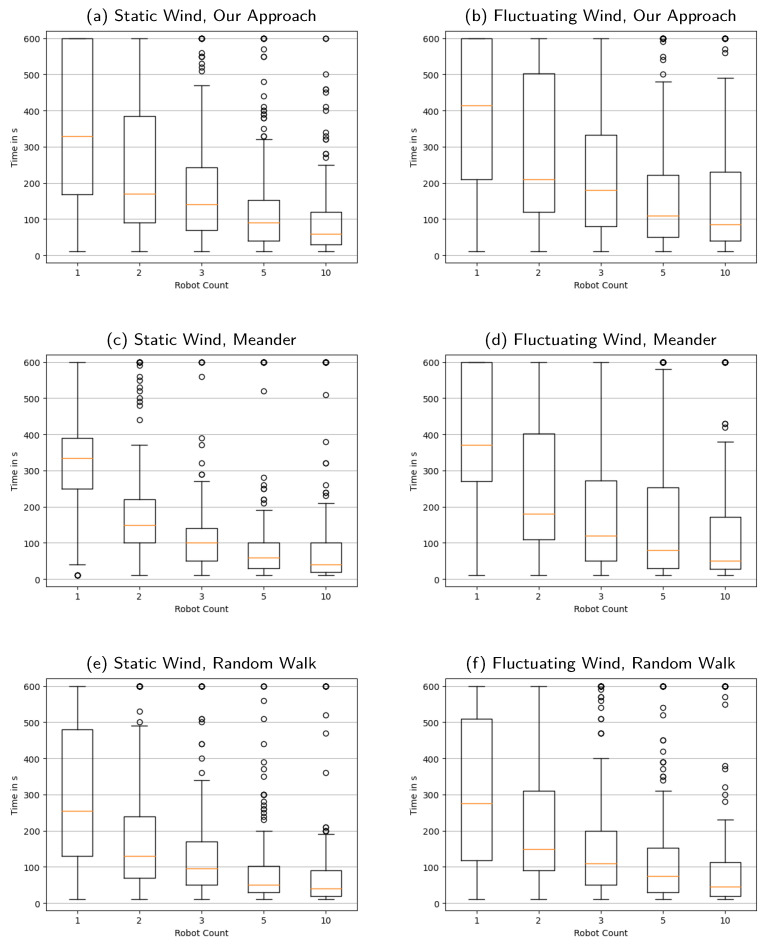
A side-by-side overview of $T_{\mathrm{first}}^{\approx }$, i.e., the distributions of the time required to reconstruct the source locations *nearly exactly*. Wind is static in the left column and fluctuating in the right. The first row presents our approach, the second is the meander benchmark, the third the random walk benchmark. Box plots show quartile boundaries, and circles indicate outliers.

**Figure A3:**
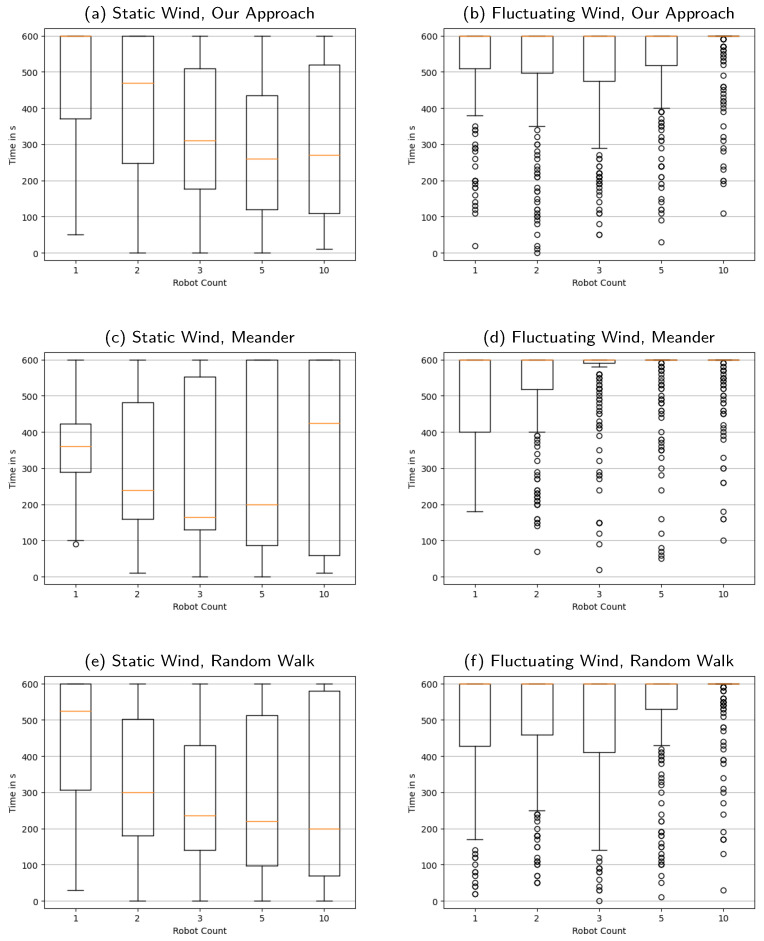
A side-by-side overview of $T_{\mathrm{last}}^{\neg \equiv }$, i.e., the distributions of the time required until the *last* reconstruction of source locations is *not exactly* correct. Wind is static in the left column and fluctuating in the right. The first row presents our approach, the second is the meander benchmark, the third the random walk benchmark. Box plots show quartile boundaries, and circles indicate outliers.

**Figure A4:**
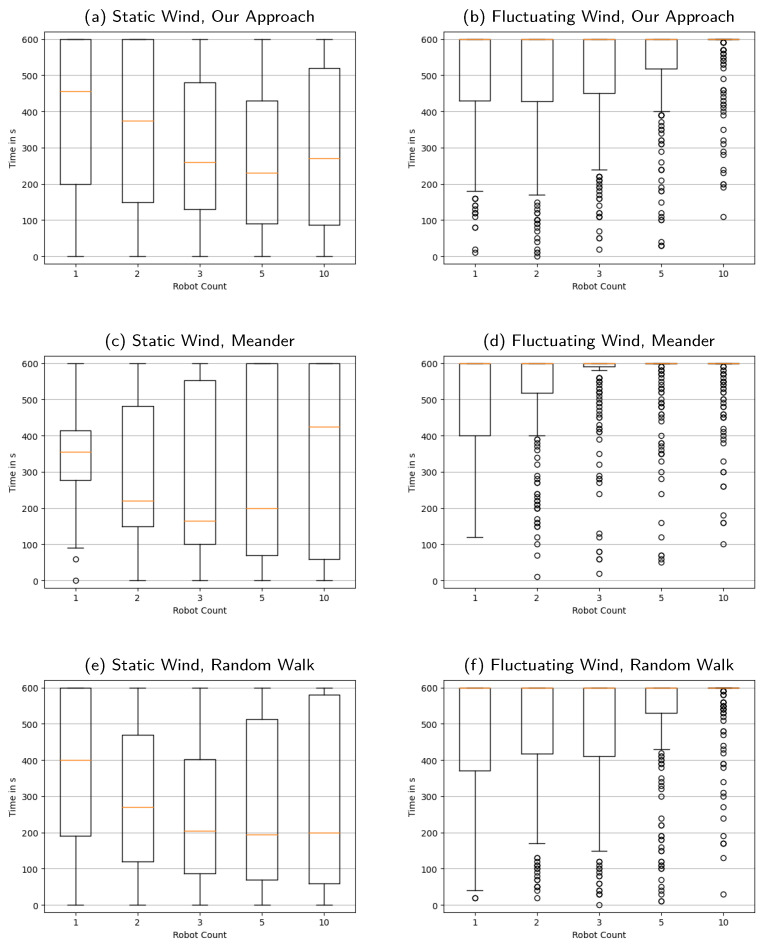
A side-by-side overview of $T_{\mathrm{last}}^{\neg \approx }$, i.e., the distributions of the time required until the *last* reconstruction of source locations is *not “nearly exactly”* correct. Wind is static in the left column and fluctuating in the right. The first row presents our approach, the second is the meander benchmark, the third the random walk benchmark. Box plots show quartile boundaries, and circles indicate outliers.

**Figure A5:**
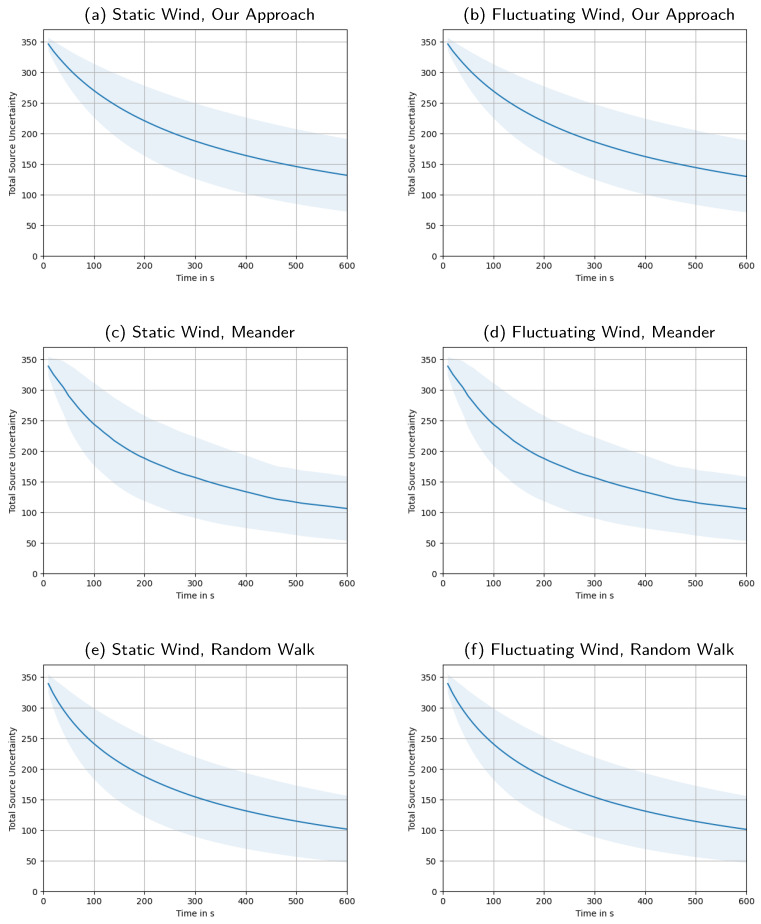
A side-by-side overview of $\mathrm{tr}(\sigma_{f_{k}}^{2})$ over time, i.e., the sum of source uncertainties at all locations, giving us a measure of the total amount of remaining uncertainty in the system. The shaded areas indicate the 1σ intervals. Wind is static in the left column and fluctuating in the right. The first row presents our approach, the second is the meander benchmark, the third the random walk benchmark.

**Figure A6:**
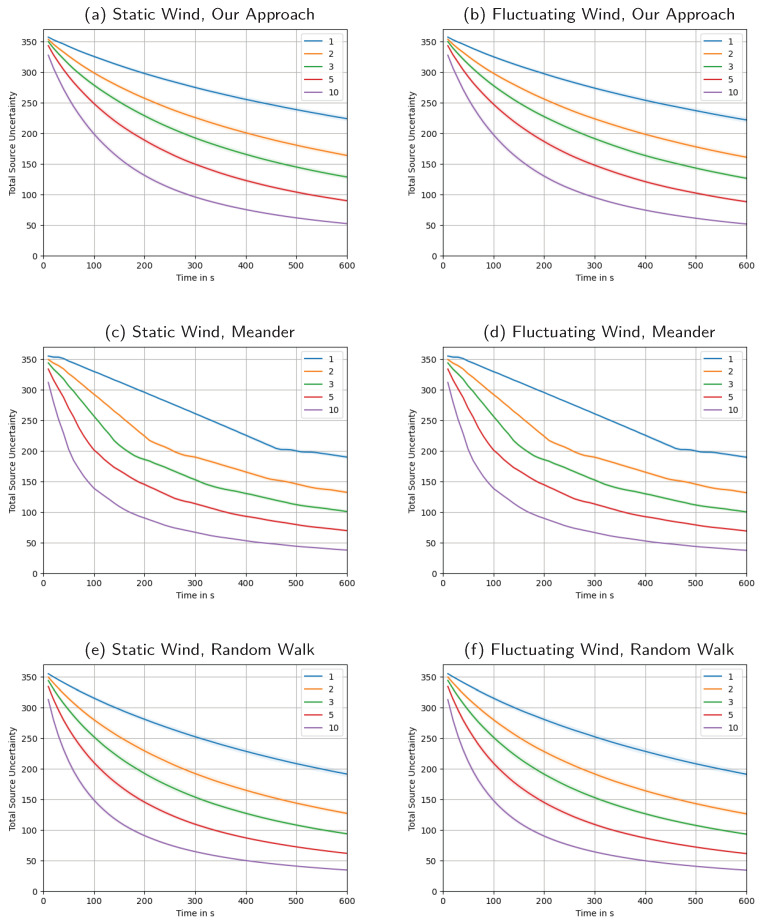
A side-by-side overview of $\mathrm{tr}(\sigma_{f_{k}}^{2})$ over time, i.e., the sum of source uncertainties at all locations, giving us a measure of the total amount of remaining uncertainty in the system. In contrast to Figure A5, averages are broken down for different swarm sizes. The shaded areas indicate the 1σ intervals. Wind is static in the left column and fluctuating in the right. The first row presents our approach, the second is the meander benchmark, the third the random walk benchmark.

Appendix A, Appendix B, Appendix C, Appendix D, Appendix E, Appendix F provide a direct side-by-side comparisons of the data presented in Figure 9, Figure 10, Figure 11, Figure 12, Figure 13 and Figure 14 for all simulations. The results of the our exploration strategy strategy are always shown in the top row of the plots in Figure A1, Figure A2, Figure A3, Figure A4, Figure A5, Figure A6, i.e., plot (a) and (b). The results of the meandering strategy can be found in the middle row, i.e., plot (c) and (d), while the results of the random walk strategy are always presented in the bottom row, i.e., plot (e) and (f). The left column (a, c, e) shows results for simulations with static wind, while on the right (b, d, f) we plots results for fluctuating wind. The same order applies for all the different metrics plotted in Appendix A, Appendix B, Appendix C, Appendix D, Appendix E, Appendix F. Please refer to the appendix when further comparisons are drawn in the following sections.

### 4.2. Our Approach vs. Meander

When comparing our approach against the meandering strategy, it is particularly useful to look at the time course of the reduction of uncertainty, as depicted in Figure A6 (Appendix F). In the meander case, plot (c), steps are visible, making evident the durations that each agent spends on their section of the map. For our approach, plot (a), the reduction in uncertainty follows a much smoother trend. With the meandering strategy, agents are constantly on the move and thus constantly measuring new locations. Also, because of the systematic nature of the trajectory, there remain no spots that are not measured. It is the combination of these reasons that lead to the fact that the final value of the total source uncertainty at t=600 s is quite a bit lower than for our approach, for all five analyzed swarm sizes. This result is not entirely surprising, as our approach, informed by the process physics, may opt to leave individual locations un-measured. Never obtaining a measurement at some locations will have an influence of the metric of $\mathrm{tr}(\sigma_{f_{k}}^{2})$, as each grid point is weighted equally in this sum; this does not necessarily mean that the reconstruction will be worse, though.

However, we see a trend that the $\mathrm{tr}(\sigma_{f_{k}}^{2})$ metric correlates with the other performances indicators like $T_{\mathrm{first}}^{\equiv }$ or $T_{\mathrm{first}}^{\approx }$, e.g., when looking at the different swarm sizes. We can observe that reducing uncertainty seems to be a good proxy for good estimation performance. This leads us to conclude that future exploration strategies can use the rate of uncertainty removal/reduction as a criterion, with the aim of achieving yet better exploration performance.

### 4.3. Our Approach vs. Random Walk

When we compare our approach to the random walk sampling strategy, we find that the random walk actually achieves significantly reduced median $T_{\mathrm{first}}^{\equiv }$ and $T_{\mathrm{first}}^{\approx }$ times. This trend holds for all swarm sizes. One particularly severe data point is the reduction in median $T_{\mathrm{first}}^{\approx }$ for the case of A=5 agents by more than a factor of 2, as can be seen in Figure A2 (Appendix B). In other cases, medians are much closer, like for A=2 in the same plots.

While it may be surprising at first that an information seeking approach would perform worse than one that relies on pure randomness, the explanation lies in the details of the strategy. In our exploration strategy, which proved effective in the static diffusion scenario as detailed in [17], agents select movement destinations guided by their expected level of informativeness. This also implies that they preferentially do not move to locations that are deemed less informative; they will even try to avoid such areas on their path towards a local extremum in uncertainty.

In fact, as a new solution is only computed every 10 s, agents may stay stationary once they have reached a local maximum in uncertainty, and only start moving towards a new location after the posterior has been updated again. While this strategy proved useful in our previous publication, here, the fact that the random walk samples more spatially distributed locations means that on average it will gather more information. Due to the fact that we have a relatively small exploration area, this means that a high information gathering rate alone, like provided by the random walk strategy, can produce already good results. This idea will be discussed further in Section 4.5.

On the basis of the probabilistic framework presented in this work, that now also incorporates advection and time-variancy, newly developed exploration strategies should take into account that greedily going for the most informative nearby measurements (like our approach does in this work) should be balanced with the total amount of sampled locations, even if each single one may be less informative. Having measurements in more different locations seems to be more important for exploration performance than more measurements in “good” locations.

When comparing the time course of the reduction in uncertainty in Appendix F, we can clearly see that the random walk is quicker at reducing the total uncertainty. This fact is most pronounced for the case of A=10 agents in the swarm. This is further evidence that moving to informative locations and stopping to collect multiple measurements there actually hinders the overall objective of dropping the total uncertainty, $\mathrm{tr}(\sigma_{f_{k}}^{2})$. It is worth emphasizing, though, that both approaches usually do find a correct “nearly exact” or exact source reconstruction easily within the simulated timeframe of 10 min (cf. Appendix A and Appendix B).

### 4.4. Fluctuating vs. Static Wind

For the discussion in the previous sections, we have kept the wind static, with the aim to achieve better comparability due to static environmental conditions. Nevertheless, we also evaluate our approach as well as all benchmark approaches against the case of a fluctuating wind model, as described in Section 3.4. The resulting plots of the simulations with varying wind are always shown in the right column in the appendix, e.g., Figure A1 (Appendix A) depicts the results of our approach in fluctuating wind in the top left plot (a), next to the plot on the top right (b), which is the same under static wind conditions.

Through the board, we can see that the fluctuating wind yields slightly reduced reconstruction performance compared to static wind. This is to be expected, as the varying wind makes it a “harder” problem. Since agents only compute a solution every 10 s, they need to average the wind vector over this time period and can thus only have an approximate process model. This reduced model fidelity presumably causes the performance drop.

Regarding the time it takes for our approach to achieve an exact ($T^{\equiv }\mathrm{first}$) or nearly exact ($T^{\approx }\mathrm{first}$) reconstruction, the median times are quite similar. However, it is worth noting that in the case of fluctuating wind, the worst-case performance worsens a bit, as evident from the upper two quartiles shown in Figure A1b and Figure A2b.

For the meandering strategy, this effect is much more pronounced, visible in the second row of Figure A1 as well as the second row of Figure A2, respectively. For fluctuating wind, plot (d), there always exists a simulation run that never finds an exact solution (i.e., $T_{\mathrm{first}}^{\equiv }\geq 600s$), independent of the swarm size. The upper quartile boundary for $T_{\mathrm{first}}^{\equiv }$ exceeds the 280 s mark across all swarm sizes.

The random walk strategy copes a lot better with fluctuating wind. While we still see some deterioration of performance when we compare the plots (e) and (f) in the last row of Figure A1 as well as Figure A2, respectively, it is much less pronounced than for the meandering benchmark. We attribute this phenomenon to the fact that the random walk, at any given moment, has the potential to explore a more widely spread-out subset of all possible source locations. Regardless of how the wind fluctuates, this provides the swarm with an improved opportunity to infer the source distribution. Overall, we can draw the conclusion that our potential field controlled robotic swarm deals with the addition of fluctuating wind in a more robust manner than the systematic sampling of the meander. The performance of the random walk also degrades a little bit, although it remains on a higher level.

One effect that we can observe throughout the board is that by introducing fluctuating wind, a once correctly identified source reconstruction may be lost again. This is visible in the boxplots for $T_{\mathrm{last}}^{\neg \equiv }$ and $T_{\mathrm{last}}^{\neg \approx }$ (right columns of Figure A3 and Figure A4), but is especially pronounced for the meander (Figure A3d). These figures show that it becomes almost an exception that a simulation run remains on the correct solution until the end of the simulation, with only a minority of total simulation runs still having an exact reconstruction at the 600 s mark. The big issue in trying to remedy this effect is, of course, that the ground truth source distribution is unknown to the swarm, and it is thus impossible to determine with certainty when the estimation has converged to the correct reconstruction. When considering possible application cases for source localization, this may not turn out to pose a problem; however, as long as at some point the correct source locations are indicated to the operators, appropriate conclusions can already been drawn, i.e., the source candidate locations can be inspected already.

### 4.5. Rate of Information Gathering vs. Size of the Explored Areal

For this work, our aim was to show that the concept introduced in [17] can be extended to other, more complex but also more realistic gas dispersion processes. Previously, we only considered a static diffusion processes. In this article, we add time-varying wind into the process description, by exploiting the advection–diffusion equation as a process model.

As we now have a time-varying process description, measurements age and get forgotten overtime. Thus, we are required to gather a lot more recent information from the environment. This circumstance led us to the decision to change the measurement acquisition process. All agents now sample the environment at a constant measurement rate of 10 Hz; in contrast, in [17] a robot just took a measurements when it reached the local minimum of the potential function, leading to a few scalar measurements every couple of seconds. Consequently, with our new measurement acquisition scheme, our rate of information gathering is much higher.

Let us illustrate: Due to the cruising speed of $0.3\;\frac{m}{s}$, a robot can theoretically cover 12 cells every 10 s. A swarm of just five agents could sample 60 in every 10 s interval. This is quite a significant proportion of the total amount of cells considering Q=361 possible source locations. So, simply by chance, even agents following a random movement strategy (i.e., the random walk) will sample enough of the environment to arrive at a reasonable reconstruction of the source distribution. This shows the limitations of the experimental setup: if the area to be explored is small enough, and if agents are able to move fast enough, they can gather enough information without an intelligent sampling strategy.

Considering the size of the area to be explored also shows another challenge. If instead of a 21 × 21 grid of cells, we chose a 101 × 101 grid, we would need to invert not only a matrix of size 1243 × 1243 (2*N* + *Q*× 2*N* + *Q*, cf. Section 2.3, page 7), but one of size 30,203 × 30,203. Considering that matrix inversions computationally scale on the order of $O(n^{3})$, this makes it computationally infeasible, especially if we intend to implement it on embedded hardware. Nevertheless, increasing the exploration area is the next step towards a realistic scenario for outdoor gas source exploration. A possible approach would be to make use of computational synergies within the swarm, since every robot comes with an onboard computer. By distributed algorithms like the message passing algorithm introduced in [13] or intelligent splitting of the area of interest, the computational complexity can be tackled in the future.

### 4.6. Performance of the Estimator

Our simulation results have shown that for the parameters of the investigated setting, a simple motion strategy is enough to achieve good source distribution reconstruction performance with the probabilistic inference framework presented in this paper. Even though the potential field control scheme adopted from our prior work is no longer the best exploration strategy, our results highlight the fact that the MAP estimator is able to recover source candidates within a few seconds. This can be seen from the boxplots of $T_{\mathrm{first}}^{\equiv }$ and $T_{\mathrm{first}}^{\approx }$ in Appendixes Appendix A and Appendix B.

A great advantage of the estimator is the fact that it relies only on a few hyper-parameters and requires little “tuning”. Next to inserting the physical parameters into the PDE model, we are left with just two parameters: a boundary weight $\lambda_{B}$ that we set to a reasonably high value, and a process weight $\lambda_{A}$, which encodes the trust into our model.

It is worth pointing out that with the estimator we presented, with a moderate swarm size of e.g., A=5 agents, we can obtain a nearly exact reconstruction of the source distribution in 120 s or less in over 50% of cases for all strategies, with or without wind. This fact can be read from Figure A2, A=5, as the boxplot quartiles below the median line all lie under the 120 s mark. Admittedly, this is being helped by the relatively small exploration area, which is at this point restricted by computational complexity. On the other hand, the source distribution may be any of the randomly drawn ones, with up to four distinct sources.

In summary, we can conclude that the probabilistic framework seems well suited to be adapted and reused, while future work should focus on developing better motion and exploration strategies.

### 4.7. Performance of the Potential Field Control

Our exploration strategy is based on a potential field based control scheme. It has been applied successfully in our previous publication (cf. [17]) with a static diffusion process, and has also shown success in reconstructing the advection–diffusion process presented in this work. However, it has not emerged as the optimal movement strategy, as it has been outperformed by benchmark approaches in certain aspects. Nevertheless, the potential field control scheme has offered us collision avoidance to ensure collision-free paths, all without the need for extensive planning efforts or parameter tuning. This alone justifies consideration for incorporating it in both current and future exploration strategies.

Also worth investigating is whether it is possible to construct a potential function that ensures a faster information gathering and better exploration performance. One limitation that makes information gathering difficult is the tendency of potential field control to adopt a “greedy” strategy. Since agents consistently follow their local gradients, they rely solely on local information without engaging in global planning. Unless the global potential field has been thoughtfully designed to account for this aspect, agents controlled by potential fields will consistently favor locally optimal solutions over globally superior ones.

## 5. Conclusions and Future Work

This work has addressed the challenging problem of estimating a gas source distribution from gas concentration measurements collected by a swarm of robots. We introduced an approach that combines a probabilistic model of the gas dispersion process based on the advection–diffusion equation with a potential field control method for information gathering with multiple mobile robots. The probabilistic framework allows to estimate the source distribution and to quantify uncertainties in the estimated parameters. The computed uncertainty map was then used to design an artificial potential function that could be utilized in a potential field control approach to guide the robots to areas of high uncertainty while avoiding mutual collisions. In addition, some novel metrics have been introduced and discussed. Having appropriate metrics is required to judge and compare exploration strategies. As it is not trivial to define what makes a *good* source reconstruction or exploration strategy, we view this discussion as a valuable contribution to the community.

Extensive simulation studies were conducted to evaluate the approach under various wind conditions, different swarm sizes, and numbers of gas sources. While the potential field based approach, which showed good performance in previous work on a static diffusion process, has proven itself successful in gathering information also in the advection–diffusion process presented here to correctly reconstruct the true source distribution, it was discovered to fall short in some aspects when compared to the benchmark strategies. That being said, it showed promise in being able to reject model mismatch, i.e., due to fluctuating wind.

Based on the analysis of the method and obtained simulation results, several key directions for future development can be identified. These include the incorporation of more realistic wind models that account for obstacles. Further, efforts should be spend on the exploration of source priors promoting sparsity, such as sparse Bayesian learning techniques. The latter are particularly attractive, as they would allow for taking into account more realistic constraints of the underlying inverse problem. Furthermore, the investigation of distributed parameter estimation approaches are of interest, similar to a distributed Kalman filter. In this way, synergies in the robotic swarm could be exploited to reduce the computational complexity in a practical realization of our GSL approach.

These avenues of research hold great promise for further advancing mobile robotic GSL, offering opportunities for enhanced accuracy and applicability in real-world scenarios.

## Data Availability

Data are contained within the article.
